# Dental Surgeons in the Army

**Published:** 1901-03

**Authors:** 


### DENTAL SURGEONS IN THE ARMY.

Attention is called to the portion, upon another page, of the new Army Bill relating to dentists in the army, and to the names of the gentlemen appointed to conduct the examinations. Comment at this stage would be injudicious, but a very large interrogation mark may be necessary in the future. At present it is the duty of all interested to give earnest support to this initial movement for the improvement of the condition of officers and men in the service.

The three men appointed have a serious responsibility, and they will be held to a strict accountability by the dental profession.

